# Notch1 pathway-mediated microRNA-151-5p promotes gastric cancer progression

**DOI:** 10.18632/oncotarget.9342

**Published:** 2016-05-13

**Authors:** Kai-Wen Hsu, Wen-Liang Fang, Kuo-Hung Huang, Tzu-Ting Huang, Hsin-Chen Lee, Rong-Hong Hsieh, Chin-Wen Chi, Tien-Shun Yeh

**Affiliations:** ^1^ Institute of Anatomy and Cell Biology, School of Medicine, National Yang-Ming University, Taipei 112, Taiwan; ^2^ Present address: Research Center for Tumor Medical Science, China Medical University, Taichung 404, Taiwan; ^3^ Institute of Clinical Medicine, School of Medicine, National Yang-Ming University, Taipei 112, Taiwan; ^4^ Department of Surgery, Taipei Veterans General Hospital, Taipei 112, Taiwan; ^5^ Department and Institute of Pharmacology, School of Medicine, National Yang-Ming University, Taipei 112, Taiwan; ^6^ School of Nutrition and Health Sciences, College of Public Health and Nutrition, Taipei Medical University, Taipei 110, Taiwan; ^7^ Department of Medical Research, Taipei Veterans General Hospital, Taipei 112, Taiwan; ^8^ Institute of Biochemistry and Molecular Biology, National Yang-Ming University, Taipei 112, Taiwan; ^9^ Genome Research Center, National Yang-Ming University, Taipei 112, Taiwan; ^10^ Graduate Institute of Medical Sciences, College of Medicine, Taipei Medical University, Taipei 110, Taiwan

**Keywords:** Notch1 receptor, miR-151, p53, FAK, gastric carcinogenesis

## Abstract

Gastric carcinoma is the third leading cause of lethal cancer worldwide. Previous studies showed that Notch1 receptor intracellular domain (N1IC), the activated form of Notch1 receptor, promotes gastric cancer progression. It has been demonstrated that a significant cross-talk interplays between Notch pathways and microRNAs (miRNAs) in controlling tumorigenesis. This study identified an intronic microRNA-151 (miR-151), which consists of two mature miRNAs, miR-151-3p and miR-151-5p, as a Notch1 receptor-induced miRNA in gastric cancer cells. Activation of Notch1 pathway enhanced expressions of miR-151 and its host gene, focal adhesion kinase (FAK), in gastric cancer cells. The levels of miR-151 in gastric cancer samples were higher than those of adjacent non-tumor samples. Activated Notch1 pathway induced CBF1-dependent FAK promoter activity. The ectopic expression of miR-151 promoted growth and progression of SC-M1 gastric cancer cells including cell viability and colony formation, migration, and invasion abilities. Activated Notch1 pathway could augment progression of gastric cancer cells through miR-151-5p and FAK. The mRNA levels of pluripotency genes, Nanog and SOX-2, tumorsphere formation ability, tumor growth, and lung metastasis of SC-M1 cells were elevated by activated Notch1 pathway through miR-151-5p. Furthermore, miR-151-5p could target 3′-untranslated region (3′-UTR) of p53 mRNA and down-regulate p53 level in SC-M1 cells. Mechanistically, Notch1/miR-151-5p axis contributed to progression of SC-M1 cells through down-regulation of p53 which in turn repressed FAK promoter activity. Taken together, these results suggest that Notch1 pathway and miR-151-5p interplay with p53 in a reciprocal regulation loop in controlling gastric carcinogenesis.

## INTRODUCTION

Gastric carcinoma is one of the most common malignant diseases and the third leading cause of cancer-related deaths in the world [1]. Notch pathways play pivotal roles in tumorigenesis [2, 3]. There are four Notch receptor paralogues (Notch1-4) and five Notch ligands in mammals [2, 3]. After binding to ligands, Notch receptors are cleaved to release Notch receptor intracellular domains, the activated forms of Notch receptors. Then Notch receptor intracellular domains are translocated into nucleus to activate expression levels of target genes *via* both C promoter binding factor-1 (CBF1)/recombination signal binding protein-Jk (RBP-Jk)-dependent and-independent pathways [2, 3]. The function of Notch pathways is complex and multi-faceted. Notch pathways act either as oncogenes or as tumor-suppressors in tumorigenesis depending on cellular context and cross-talk with other pathways [2, 3]. In gastric cancer cells, Notch1 and Notch2 pathways have been shown to promote tumorigenesis [4, 5]. Furthermore, Notch3 receptor expression was associated with gastric cancer development [6] and Notch4 receptor promoted gastric cancer growth [7].

Mounting evidence demonstrates that microRNAs (miRNAs) act either as oncogenes or as tumor-suppressors in development and progression of tumors [8]. miRNAs are small non-coding RNAs binding to the 3′-untranslated regions (3′-UTRs) of target mRNAs and regulate several biological processes [8, 9]. Several Notch-associated miRNAs have been identified in cancers revealing a significant cross-talk between Notch pathways and miRNAs in tumorigenesis. For example, miR-34 family inhibited Notch1 and Notch2 levels in glioma [10] and gastric cancer [11] cells and suppressed self-renewal of pancreatic cancer stem cells through targeting Notch1 and Notch2 receptors [12]. Additionally, Notch1 receptor interplayed with several miRNAs in cancer cells [13]. There were reciprocal regulation loops between Notch2 pathway and miR-205 [14] as well as miR-23b [15] in controlling mammary stem cell fate and gastric carcinogenesis, respectively. Notch3 receptor regulated miR-223 level in T-cell acute lymphoblastic leukemia [16]. In the present study, we identified miR-151 derived from the intron of focal adhesion kinase (FAK) gene [17] as a Notch1 receptor-associated miRNA and delineated its role in a reciprocal regulation loop of gastric carcinogenesis.

## RESULTS

### Activated Notch1 pathway enhanced miR-151 and FAK expressions in gastric cancer cells

**Figure 1:**
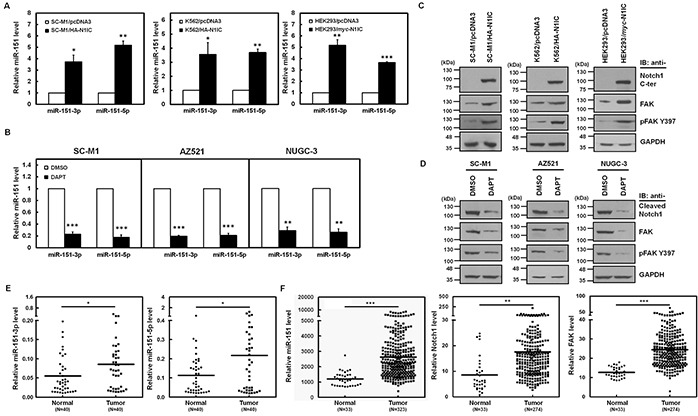
Activated Notch1 pathway enhanced miR-151 and FAK expressions in gastric cancer cells **A.** The relative levels of miR-151-3p and miR-151-5p in N1IC-expressing SC-M1/HA-N1IC (*left*), K562/HA-N1IC (*middle*), and HEK293/myc-N1IC (*right*) cells and their control cells (SC-M1/pcDNA3, K562/pcDNA3, and HEK293/pcDNA3 cells, respectively) were measured by miRNA quantitative real-time PCR. Levels of miR-151-3p and miR-151-5p in control cells were set to unity. **B.** The relative levels of miR-151-3p and miR-151-5p in SC-M1, AZ521, and NUGC-3 cells were determined using miRNA quantitative real-time PCR after treatment with 50 mM DAPT for 24 hours. **C.** Whole-cell extracts of N1IC-expressing SC-M1/HA-N1IC (*left*), K562/HA-N1IC (*middle*), and HEK293/myc-N1IC (*right*) cells and their control cells were prepared and then analyzed by Western blot analysis using anti-Notch1 C-terminal (C-ter), anti-FAK, anti-pFAK Y397, and anti-GAPDH antibodies. **D.** After treated with 50 mM DAPT for 24 hours, whole-cell extracts of SC-M1 (*left*), AZ521 (*middle*), and NUGC-3 (*right*) cells were prepared for Western blot analysis using anti-cleaved Notch1, anti-FAK, anti-pFAK Y397, and anti-GAPDH antibodies. **E.** Tumor and the adjacent non-tumor tissue sample pairs from gastric cancer patients (n=40) were examined using miRNA quantitative real-time PCR analysis. Levels of miR-151-5p and miR-151-3p in the gastric cancer tissues were compared with those of the corresponding adjacent normal tissues. **F.** Data of level 3 of mRNA and miRNA expressions from stomach adenocarcinoma samples and normal counterparts were downloaded from the TCGA and Broad GDAC Firehose data portal. Both mRNA RPKM (Reads per Kilobase of exon model per Million) and microRNA reads per million mappable reads of all samples were selected and analyzed to compare abundances using GraphPad Prism 5 software. The transcript levels of miR-151, Notch1 receptor, and FAK in stomach adenocarcinoma samples (miR-151, n=323; Notch1 receptor and FAK, n=274) and normal tissue samples (n=33) were measured by RNA sequencing in TCGA data. *, *P* <0.05; **, *P* <0.01; ***, *P* <0.001.

To identify the Notch1 receptor-induced miRNAs in gastric cancer cells, miRNA quantitative real-time PCR analyses were performed in Notch1 receptor intracellular domain (N1IC)-expressing SC-M1 (SC-M1/HA-N1IC) cells and control cells. SC-M1 cells, human stomach adenocarcinoma cells, were used herein because more than 95% of tumors of stomach are adenocarcinomas. An intronic microRNA miR-151, which consists of miR-151-3p and miR-151-5p, was identified and further confirmed to be the potent Notch1 pathway-inducing miRNA (Figure 1A, *left*). The miR-151 levels were also elevated in N1IC-expressing K562 (K562/HA-N1IC) and HEK293 (HEK293/myc-N1IC) cells (Figure 1A, *middle* and *right*). To confirm the activated Notch-mediated miR-151 expression, levels of miR-151 in SC-M1, AZ521, and NUGC-3 gastric cancer cells were examined by analysis of miRNA quantitative real-time PCR in the presence of g-secretase inhibitor N-[N-(3,5-Difluorophenacetyl)-L-alanyl]-S-phenylglycine *t*-butyl ester (DAPT). Both miR-151-3p and miR-151-5p expressions were suppressed by DAPT treatment in these cells (Figure 1B). Because the intronic miR-151 is often expressed together with its host gene encoding FAK [17], we also analyzed whether activated Notch1 pathway promotes FAK expression and the phosphorylated state of Tyr397 residue in FAK (referred to hereafter as pFAK Y397), a pivotal event in the FAK-mediated pathway. Western blot analyses demonstrated that FAK and pFAK Y397 levels were increased by N1IC in SC-M1/HA-N1IC, K562/HA-N1IC, and HEK293/myc-N1IC cells (Figure 1C). In contrast, their protein levels were decreased in SC-M1, AZ521, and NUGC-3 cells treated with DAPT (Figure 1D).

The miR-151 gene is localized to chromosome 8q which is frequently amplified in cancers [18–24] including gastric cancer. To examine the clinical relevance of miR-151-3p and miR-151-5p expressions, the miRNA quantitative real-time PCR was employed on gastric cancer samples and the corresponding adjacent normal tissues of gastric cancer patients. Levels of miR-151-3p (Figure 1E, *left*) and miR-151-5p (Figure 1E, *right*) in tumor samples were higher than those of adjacent non-tumor samples. Data obtained from The Cancer Genome Atlas (TCGA) were also analyzed and found that levels of miR-151 and Notch1 and FAK mRNAs were significantly increased in majority of stomach adenocarcinoma samples as compared with normal tissue samples (Figure 1F).

### Activated Notch1 pathway induced FAK promoter activity through CBF1

**Figure 2:**
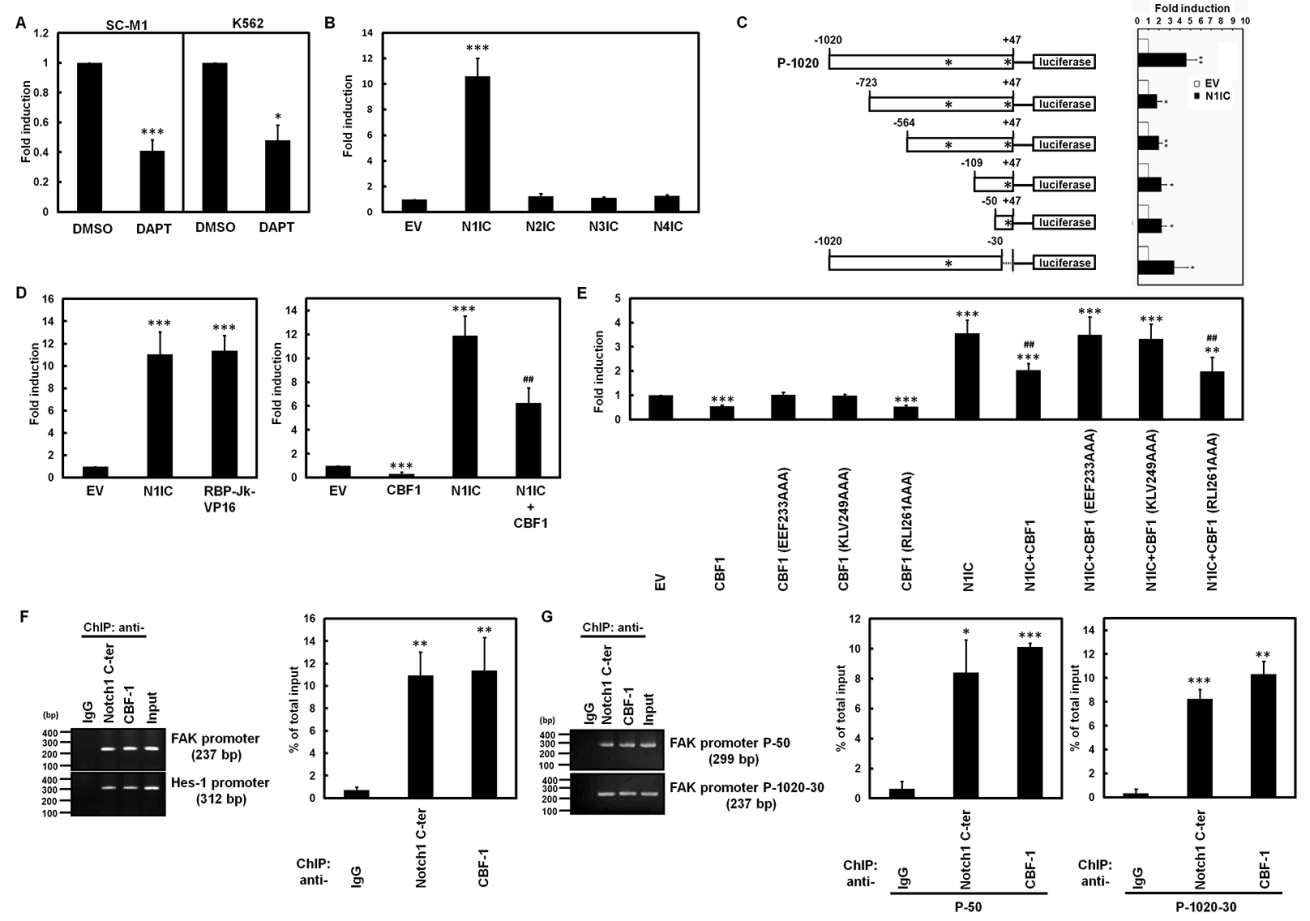
Activated Notch1 pathway induced FAK promoter activity through CBF1 **A.** After transfection with reporter plasmid P-1020 containing FAK promoter (nucleotide −1,020 to +47) into SC-M1 and K562 cells, the transfected cells were treated with 50 mM DAPT for 48 hours and then used for reporter gene assay. **B.** Reporter plasmid P-1020 was co-transfected with expression constructs of Notch1 receptor (N1IC), Notch2 receptor (N2IC), Notch3 receptor (N3IC), and Notch4 receptor (N4IC) intracellular domains or empty vector (EV) into SC-M1 cells for 48 hours for reporter gene assay. **C.** Schematic representation of luciferase reporter plasmids (P-50, P-109, P-564, P-723, P-1020-30, and P-1020) containing various lengths of human FAK promoter (*left*). Stars indicate positions of the putative CBF1-response elements. Reporter plasmids containing various lengths of FAK promoter were co-transfected with N1IC-expressing construct into K562 cells for reporter gene assay (*right*). **D.** Reporter plasmid P-1020 was co-transfected with expression constructs of N1IC and constitutively active RBP-Jk-VP16 fusion protein (*left*) or CBF1 (*right*) into SC-M1 cells for reporter gene assay. **E.** Reporter plasmid P-1020 was co-transfected with expression constructs of N1IC, wild-type CBF1, and CBF1 mutants including EEF233AAA, KLV249AAA, and RLI261AAA into K562 cells for reporter gene assay. **F.** SC-M1/HA-N1IC cells were harvested for ChIP assay using anti-IgG, anti-Notch1 C-ter, and anti-CBF1 antibodies. The immunoprecipitated DNAs were used to amplify PCR products of FAK and Hes-1 promoters (*left*). Percentages of immunoprecipitated DNAs were quantified by quantitative real-time PCR and then normalized to total input DNA (*right*). **G.** After transfection with reporter plasmids P-50 and P-1020-30 for 48 hours, the transfected K562/HA-N1IC cells were harvested for ChIP assay as described above. The immunoprecipitated DNAs were used to amplify the PCR products in the region of FAK promoter in P-50 and P-1020-30 reporter plasmids. *, *P* <0.05; **, *P* <0.01; ***, *P* <0.001. ##, *P* < 0.01; ###, *P*< 0.001.

To investigate whether Notch pathways induce miR-151 and FAK expressions through FAK promoter, reporter plasmid containing the human FAK promoter (nucleotide −1,020 to +47) was transfected into SC-M1 and K562 cells for reporter gene assay in the presence or absence of DAPT (Figure 2A). The reporter assay showed that FAK promoter activities were inhibited by DAPT treatment in both cells. Furthermore, only the exogenous N1IC significantly enhanced FAK promoter activity after co-transfection with constructs of the four Notch receptor intracellular domains in SC-M1 (Figure 2B) and K562 cells (Supplementary Figure S1A). To map the critical regions of FAK promoter transactivated by N1IC, the reporter plasmids containing different regions of FAK promoter were co-transfected with N1IC-expressing construct into K562 cells for reporter gene assay (Figure 2C). Even narrow the FAK promoter region down to nucleotide −50 to +47 was still sufficient for the N1IC-mediated transactivation.

There are two putative CBF1-binding sites in FAK promoter (Figure 2C). To clarify whether the N1IC-induced FAK promoter activity depends on CBF1, reporter gene assay was performed after co-transfection of reporter plasmid containing FAK promoter with expression constructs of wild-type CBF1 or the constitutively active RBP-Jκ-VP16 fusion protein. FAK promoter activity was enhanced by RBP-Jκ-VP16 fusion protein (Figure 2D, *left* and Supplementary Figure S1B, *left*) but suppressed by wild-type CBF1 (Figure 2D, *right* and Supplementary Figure S1B, *right*). The N1IC-induced FAK promoter activity was also attenuated by wild-type CBF1 (Figure 2D, *right* and Supplementary Figure S1B, *right*). Additionally, reporter gene assays were performed after co-transfection of reporter plasmid containing FAK promoter with expression constructs of wild-type CBF1 and CBF1 mutants including RLI261AAA expressing normal nuclear staining and EEF233AAA as well as KLV249AAA expressing cytosolic staining [25]. The nuclear-located RLI261AAA mutant but not cytosolic-located EEF233AAA or KLV249AAA mutants suppressed FAK promoter activity and attenuated N1IC-induced FAK promoter activity (Figure 2E).

We surmised that N1IC and CBF1 might bind to the DNA of FAK promoter to activate reporter gene activity in the context of living cells. The DNA-binding abilities of N1IC and CBF1 on FAK promoter in N1IC-expressing SC-M1/HA-N1IC (Figure 2F) and K562/HA-N1IC (Supplementary Figure S1C) cells were examined by the chromatin immunoprecipitation (ChIP) assay using anti-Notch1 C-terminal and anti-CBF1 antibodies. The ChIP assay showed that N1IC and CBF1 bound to promoters of FAK and a target of CBF1-dependent Notch1 pathway, Hes-1, in the chromosomal DNAs of SC-M1/HA-N1IC and K562/HA-N1IC cells. Moreover, N1IC and CBF1 also bound to the FAK promoter region of reporter plasmid in K562/HA-N1IC cells that were transfected with reporter plasmids containing FAK promoter by ChIP assay (Figure 2G). These results clearly indicated that Notch1 pathway activated FAK promoter activity through a CBF1-dependent manner.

### miR-151 promoted growth and progression of SC-M1 cells

**Figure 3:**
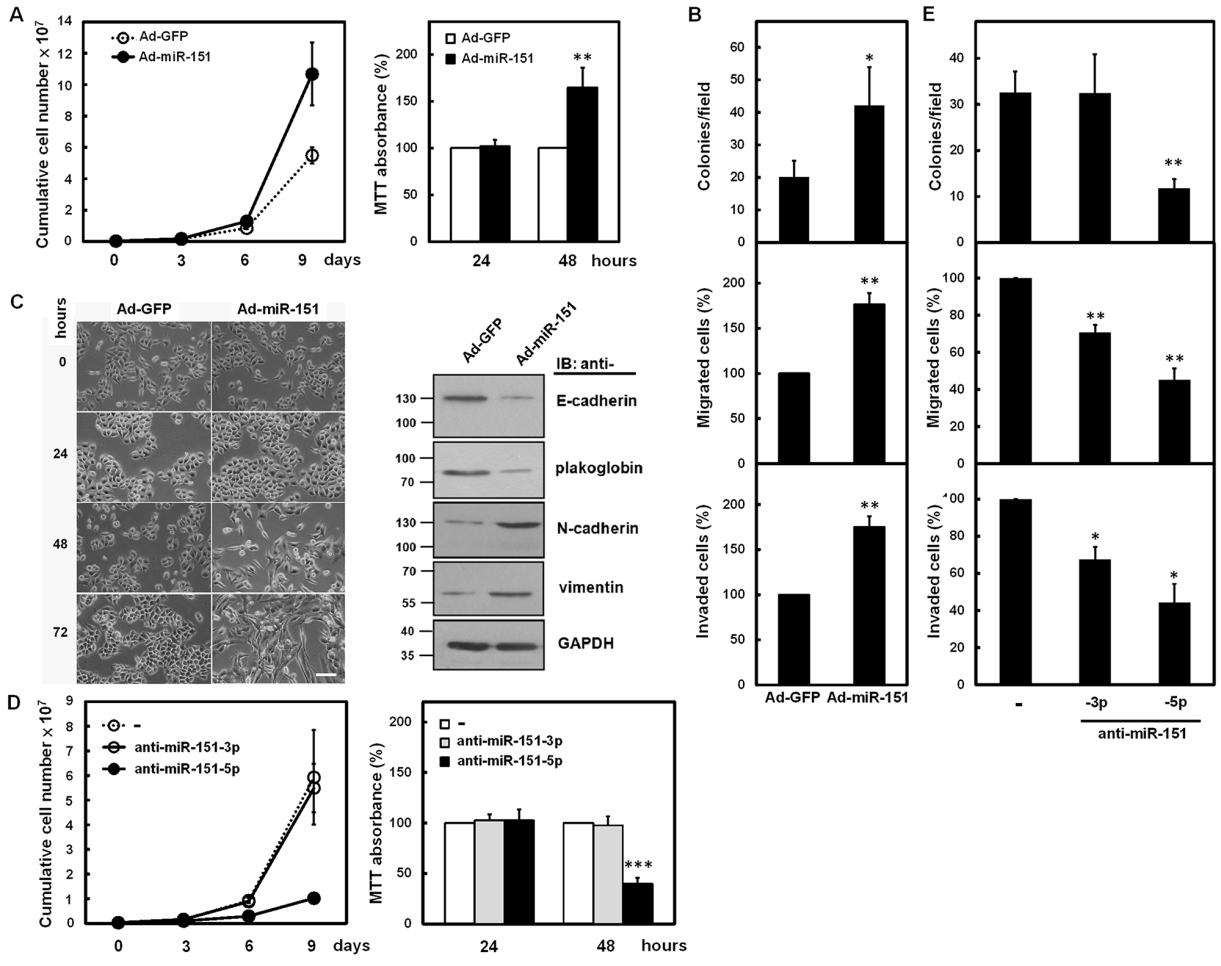
miR-151 promoted growth and progression of SC-M1 cells **A-C.** SC-M1 cells were infected with adenoviruses expressing miR-151 (Ad-miR-151) or GFP (Ad-GFP). The infected cells were seeded and then counted by trypan blue exclusion method (*left*) or incubated to analyze cell proliferation by MTT assay (*right*) at the time indicated (A). The infected cells were seeded for colony formation (*upper*), migration (*middle*), and invasion (*lower*) assays (B). Means of at least three independent experiments performed in triplicate are shown. The infected cells were also seeded onto 6-well plates for morphological examination (*left*) at the time indicated (C). Bar, 100 μm. Whole-cell extracts of SC-M1 cells after infection for 48 hours were prepared for Western blot analysis using anti-E-cadherin, anti-plakoglobin, anti-N-cadherin, anti-vimentin, and anti-GAPDH antibodies (*right*). **D, E.** SC-M1 cells transfected with 100 nM antagomir-151-3p (anti-miR-151-3p) or antagomir-151-5p (anti-miR-151-5p) were seeded and subsequently counted by trypan blue exclusion method (*left*) or incubated to analyze cell proliferation by MTT assay (*right*) at the time indicated (D). The infected cells were also seeded for colony formation (*upper*), migration (*middle*), and invasion (*lower*) assays (E). Means of at least three independent experiments performed in triplicate are shown. *, *P* <0.05; **, *P* <0.01; ***, *P* <0.001.

To investigate the effect of miR-151 on growth and progression of gastric cancer cells, the adenoviral system exogenously expressing miR-151 was established. The miRNA quantitative real-time PCR analysis (Supplementary Figure S2A) showed that levels of miR-151-3p and miR-151-5p were increased in SC-M1 cells infected with miR-151-expressing adenoviruses as compared with those infected with green fluorescent protein (GFP)-expressing adenoviruses. The cumulative cell numbers of miR-151-expressing adenoviruses-infected SC-M1 cells were further elevated than those of control adenoviruses-infected cells after 9 days by trypan blue exclusion method (Figure 3A, *left*). Results of 3-(4,5-dimethyl-2-thiazolyl)-2,5-diphenyl tetrazolium bromide (MTT) assay showed that growth of SC-M1 cells was enhanced after infection with miR-151-expressing adenoviruses for 48 hours (Figure 3A, *right*). The colony formation, migration, and invasion abilities of SC-M1 cells were also found to be elevated after miR-151 overexpression in SC-M1 cells (Figure 3B).

Next, we analyzed whether miR-151 regulates epithelial-mesenchymal transition (EMT) of gastric cancer cells. SC-M1 cells grew dispersedly and displayed a spindle- and fibroblast-like morphology after infection with miR-151-expressing adenoviruses for 48 or 72 hours (Figure 3C, *left*). Western blot analyses showed that the epithelial markers E-cadherin and plakoglobin were down-regulated, in contrary, the mesenchymal markers N-cadherin and vimentin were up-regulated in SC-M1 cells infected with miR-151-expressing adenoviruses (Figure 3C, *right*).

To address whether miR-151-3p or miR-151-5p exert the main effect on increment of gastric cancer progression, antagomir-151-3p and antagomir-151-5p reagents, chemically modified antisense RNA oligonucleotides, were employed to inhibit the function of endogenous miR-151. The miRNA quantitative real-time PCR showed that transient transfection with 100 nM of antagomir-151-3p and antagomir-151-5p into SC-M1 cells significantly knocked down miR-151-3p and miR-151-5p expressions, respectively, in SC-M1 cells (Supplementary Figure S2B). Cell growth determined by trypan blue (Figure 3D, *left*) or MTT (Figure 3D, *right*) assays and colony formation abilities (Figure 3E, *upper*) in SC-M1 cells were suppressed after transfection with antagomir-151-5p but not antagomir-151-3p. The migration and invasion abilities were also significantly inhibited after transfection with antagomir-151-5p and slightly suppressed after transfection with antagomir-151-3p (Figure 3E, *middle* and *lower*).

### Activated Notch1 pathway promoted gastric cancer progression through FAK

It has been demonstrated that FAK activation promotes gastric cancer progression [26]. To explore whether FAK involves in N1IC-enhanced gastric cancer progression, the endogenous FAK was knocked down by small interfering RNA (siRNA) vectors against FAK in SC-M1 cells (Supplementary Figure S3A). The enhanced abilities of N1IC on colony formation, migration, and invasion of SC-M1/HA-N1IC cells were suppressed after transfection with siRNA vectors against FAK (Supplementary Figure S3B).

### N1IC augmented gastric cancer progression through miR-151-5p

**Figure 4:**
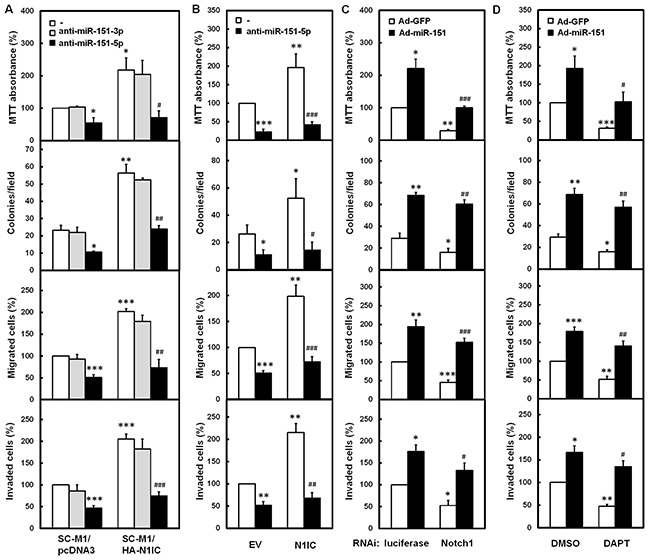
N1IC augmented gastric cancer progression through miR-151-5p **A.** The N1IC-expressing SC-M1/HA-N1IC and SC-M1/pcDNA3 control cells were transfected with 100 nM antagomir-151-3p (anti-miR-151-3p), antagomir-151-5p (anti-miR-151-5p), or scrambled control (−) and then seeded for MTT, colony formation, migration, and invasion assays. **B.** SC-M1 cells were co-transfected with N1IC-expressing construct (N1IC) or empty vector (EV) and 100 nM antagomir-151-5p or scrambled control and then seeded for MTT, colony formation, migration, and invasion assays. **C.** After transfection with siRNA vectors against Notch1 receptor or luciferase, SC-M1 cells were infected with adenoviruses expressing miR-151 (Ad-miR-151) or GFP (Ad-GFP) and then seeded for MTT, colony formation, migration, and invasion assays. **D.** After infected with adenoviruses expressing miR-151 or GFP, SC-M1 cells were treated with 50 mM DAPT and then seeded for MTT, colony formation, migration, and invasion assays. *, *P* <0.05; **, *P* <0.01; ***, *P* <0.001. #, *P* < 0.05; ##, *P* < 0.01; ###, *P*< 0.001.

To further investigate whether N1IC promotes gastric cancer progression through miR-151, N1IC-expressing SC-M1/HA-N1IC cells and SC-M1/pcDNA3 control cells were transfected with antagomir-151-3p or antagomir-151-5p to knock down miR-151. The cell growth, colony formation, migration, and invasion abilities of SC-M1/pcDNA3 control cells were suppressed after transfection with antagomir-151-5p, but not antagomir-151-3p (Figure 4A). Moreover, the N1IC-enhanced ability on cancer progression of SC-M1/HA-N1IC cells was abolished after transfected with antagomir-151-5p, but not with antagomir-151-3p. The N1IC-promoted gastric cancer progression of SC-M1 cells was also alleviated by miR-151-5p knockdown after co-transfection with N1IC-expressing construct and antagomir-151-5p (Figure 4B). Furthermore, the suppression of cancer progression by Notch1 knockdown could be rescued by miR-151 overexpression (Figure 4C). The suppression of cancer progression by DAPT treatment could also be restored with miR-151-expressing adenoviruses infection (Figure 4D).

### N1IC enhanced progression of AGS, KATO III, and NUGC-3 gastric cancer cells through miR-151-5p

Western blot analysis and miRNA quantitative real-time PCR were performed to analyze the relative levels of N1IC, FAK, and miR-151 in gastric cancer cells including SC-M1, AGS, AZ521, NUGC-3, KATO III, NCI-N87, and SNU-16 cells. The cleaved Notch1 receptors were detectable in SC-M1, AZ521, NUGC-3, and NCI-N87 cells, whereas negatively or weakly expressed in AGS, KATO III, and SNU-16 cells (Supplementary Figure S4A). FAK expressions were also negative or weak in AGS, KATO III, and SNU-16 cells. Levels of miR-151-5p were higher in NUGC-3 and NCI-N87 cells, while weakly expressed in AGS, KATO III, and SNU-16 cells (Supplementary Figure S4B). Expressions of miR-151-3p were slightly higher in NCI-N87 cells, while weakly expressed in other cells.

To study the effect of Notch1/miR-151-5p axis on gastric cancer progression, colony formation, migration, and invasion abilities of AGS and KATO III cells, which scarcely expressed the cleaved Notch1 receptor, were evaluated after co-transfection of N1IC-expressing construct and antagomir-151-5p. The enhanced abilities of N1IC on cancer progression of AGS (Supplementary Figure S4C) and KATO III (Supplementary Figure S4D) cells were attenuated by miR-151-5p knockdown. Furthermore, the reduced progression abilities by Notch1 knockdown (Supplementary Figure S4E) or DAPT treatment (Supplementary Figure S4F) were restored after infection with miR-151-expressing adenoviruses in NUGC-3 cells, the cell with detectable cleaved Notch1 receptor.

### N1IC elevated ability of tumorsphere formation of SC-M1 cells through miR-151-5p

**Figure 5:**
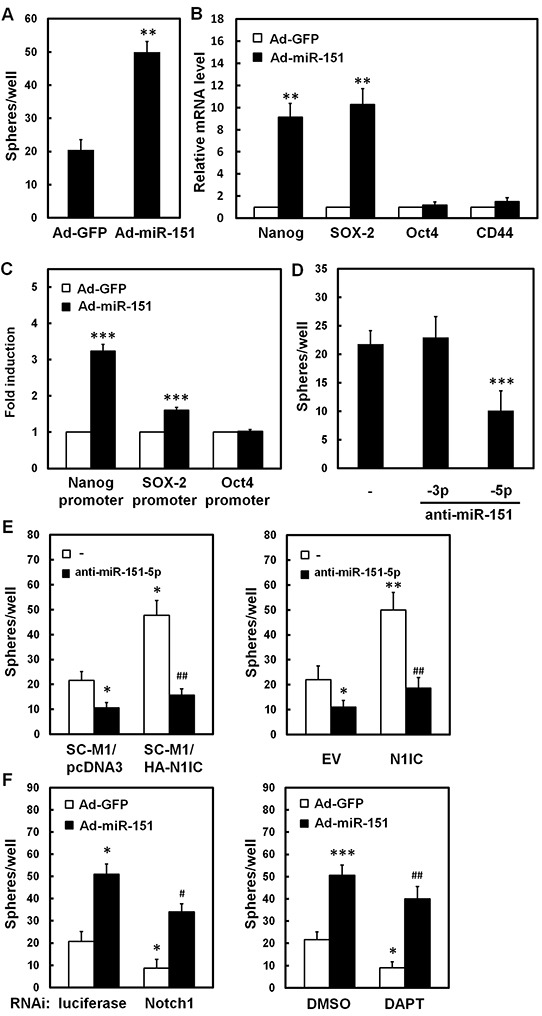
N1IC elevated ability of tumorsphere formation of SC-M1 cells through miR-151-5p **A, B.** SC-M1 cells were infected with adenoviruses expressing miR-151 (Ad-miR-151) or GFP (Ad-GFP). Then the infected cells were seeded onto 24-well ultra-low attachment plates under stem cell-selective conditions for subsequent tumorsphere formation assay (A). The transcript levels of Nanog, SOX-2, Oct4, and CD44 in the infected cells were also determined by quantitative real-time PCR (B). **C.** After transfection with reporter plasmids containing Nanog, SOX-2, and Oct4 promoters, SC-M1 cells were infected with adenoviruses expressing miR-151 or GFP for subsequent reporter gene assay. **D.** SC-M1 cells transfected with 100 nM antagomir-151-3p (anti-miR-151-3p), antagomir-151-5p (anti-miR-151-5p), or scrambled control (−) were seeded for tumorsphere assay. **E.** The N1IC-expressing SC-M1/HA-N1IC and SC-M1/pcDNA3 control cells were transfected with 100 nM antagomir-151-5p or scrambled control for tumorsphere assay (*left*). SC-M1 cells were co-transfected with N1IC-expressing construct (N1IC) or empty vector (EV) and 100 nM antagomir-151-5p or scrambled control for tumorsphere assay (*right*). **F.** After transfection with siRNA vectors against Notch1 receptor or luciferase, SC-M1 cells were infected with adenoviruses expressing miR-151 or GFP and then seeded for tumorsphere formation assay (*left*). After infected with adenoviruses expressing miR-151 or GFP, SC-M1 cells were also treated with 50 mM DAPT for tumorsphere formation assay (*right*). *, *P* <0.05; **, *P* <0.01; ***, *P* <0.001. #, *P* < 0.05; ##, *P* < 0.01.

The ability of miR-151 in maintenance of cancer stem-like phenotype of SC-M1 cells was analyzed by tumorsphere formation assay. The tumorsphere formation ability was elevated in SC-M1 cells after miR-151-expressing adenoviruses infection (Figure 5A). The quantitative real-time PCR analyses showed that mRNA levels of pluripotency genes Nanog and SOX-2, but not Oct4 and CD44, were significantly increased by miR-151 overexpression in SC-M1 cells (Figure 5B). The activities of reporter genes containing promoters of Nanog and SOX-2 but not of Oct4 were increased by miR-151 overexpression (Figure 5C and Supplementary Figure S5). Additionally, tumorsphere formation ability of SC-M1 cells was repressed after transfection with antagomir-151-5p, but not with antagomir-151-3p (Figure 5D). The increment of tumorsphere formation abilities in N1IC-expressing SC-M1/HA-N1IC cells (Figure 5E, *left*) and SC-M1 cells transfected with N1IC-expressing construct (Figure 5E, *right*) was suppressed after transfection with antagomir-151-5p. The reduction of tumorsphere formation ability of SC-M1 cells by Notch1 receptor knockdown (Figure 5F, *left*) or DAPT treatment (Figure 5F, *right*) could be restored by miR-151 overexpression.

### N1IC promoted tumor growth and lung metastasis of SC-M1 cells through miR-151-5p *in vivo*

**Figure 6:**
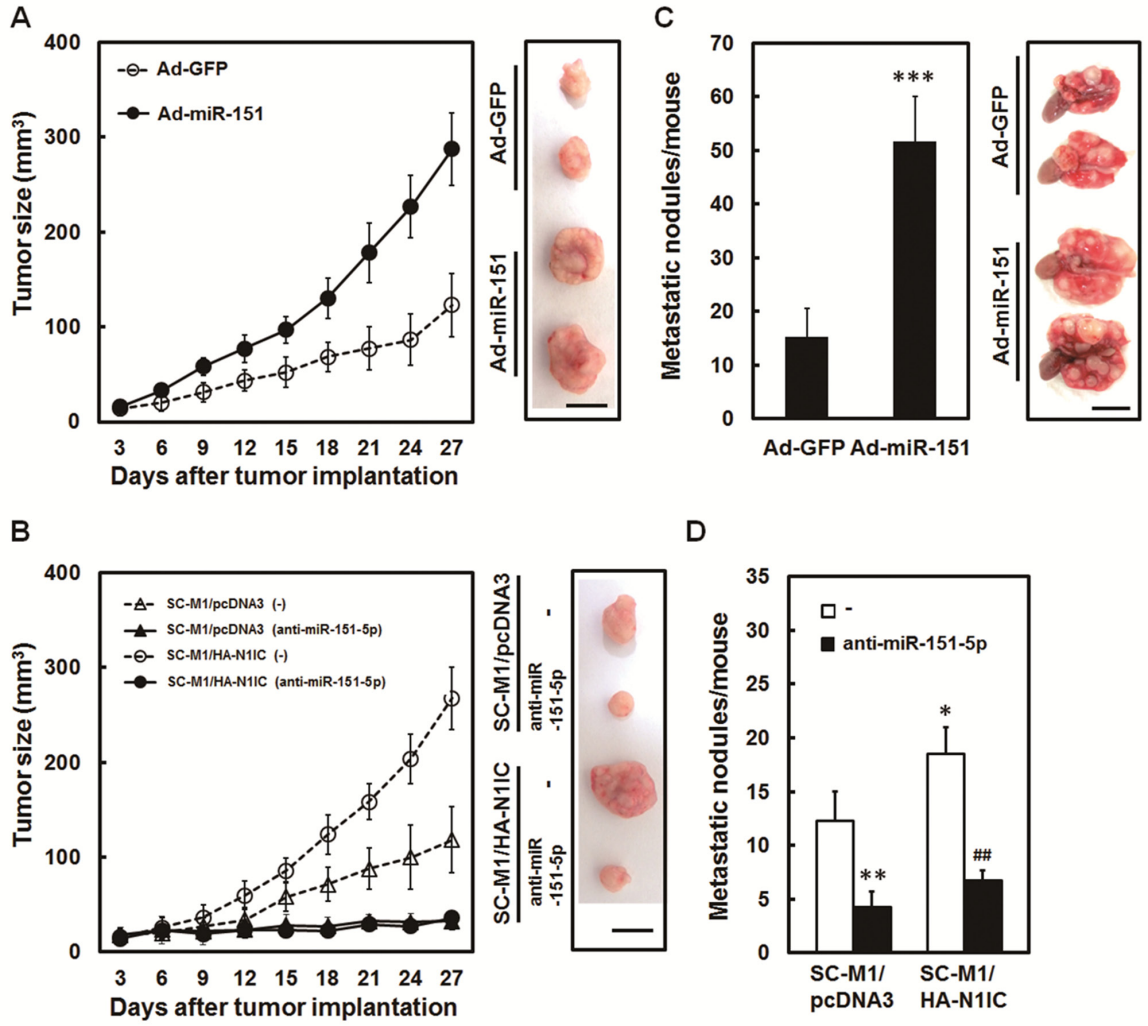
N1IC promoted tumor growth and lung metastasis of SC-M1 cells through miR-151-5p *in vivo* **A.** After infection with adenoviruses expressing miR-151 (Ad-miR-151) or GFP (Ad-GFP), SC-M1 cells were subcutaneously injected into nude mice (*n* = 6 per group) for measurement of tumor sizes at the time indicated. On day 27, the mice were sacrificed and then subcutaneous tumors were excised. Data are representative of three experiments. Bar, 1.0 cm. **B.** After transfection with 100 nM antagomir-151-5p (anti-miR-151-5p) or scrambled control (−), The N1IC-expressing SC-M1/HA-N1IC and SC-M1/pcDNA3 control cells were subcutaneously injected into nude mice (*n* = 5 per group) for measurement of tumor sizes at the time indicated. The mice were sacrificed on day 27 and subsequently subcutaneous tumors were excised. Data are representative of three experiments. Bar, 1.0 cm. **C.** After infection with adenoviruses expressing miR-151 or GFP, SC-M1 cells were injected into NOD-SCID mice (*n* = 7 per group) by tail vein injection for measurement of metastatic nodules in lungs. After 18 weeks, the mice were sacrificed and the metastatic nodules in the lungs were counted by gross and microscopic examination. Data are from a representative experiment that was performed three times with identical results. **D.** After transfection with 100 nM antagomir-151-5p or scrambled control, SC-M1/HA-N1IC or SC-M1/pcDNA3 cells were injected into NOD-SCID mice (*n* = 5 per group) by tail vein injection for measurement of metastatic nodules in lungs. The mice were sacrificed after 18 weeks and the metastatic nodules in the lungs were counted by gross and microscopic examination. Data are from a representative experiment that was performed three times with identical results. *, *P* <0.05; **, *P* <0.01; ***, *P* <0.001. ##, *P* < 0.01.

Mice model was employed to further assess roles of miR-151-5p in tumor growth and metastatic colonization of gastric cancer cells. Nude mice were subcutaneously injected with miR-151-expressing adenoviruses-infected SC-M1 cells to analyze the effect of miR-151 on the xenografted tumor growth. Tumor sizes of SC-M1 cells were augmented by miR-151 overexpression (Figure 6A). The N1IC-elevated tumor growth of SC-M1/HA-N1IC cells was suppressed by antagomir-151-5p transfection (Figure 6B). The miR-151-expressing adenoviruses-infected SC-M1 cells were intravenously injected into lateral tail vein of non-obese diabetic severe-combined immunodeficiency (NOD-SCID) mice. After 18 weeks, the numbers of metastatic nodules in lungs were significantly increased in mice injected with SC-M1 cells overexpressing miR-151 (Figure 6C). The N1IC-enhanced ability in forming metastatic nodules in lungs of SC-M1/HA-N1IC cells-injected mice could be suppressed by miR-151-5p knockdown (Figure 6D).

### Notch1/miR-151-5p axis elevated aggressiveness of SC-M1 cells through p53 down-regulation

**Figure 7:**
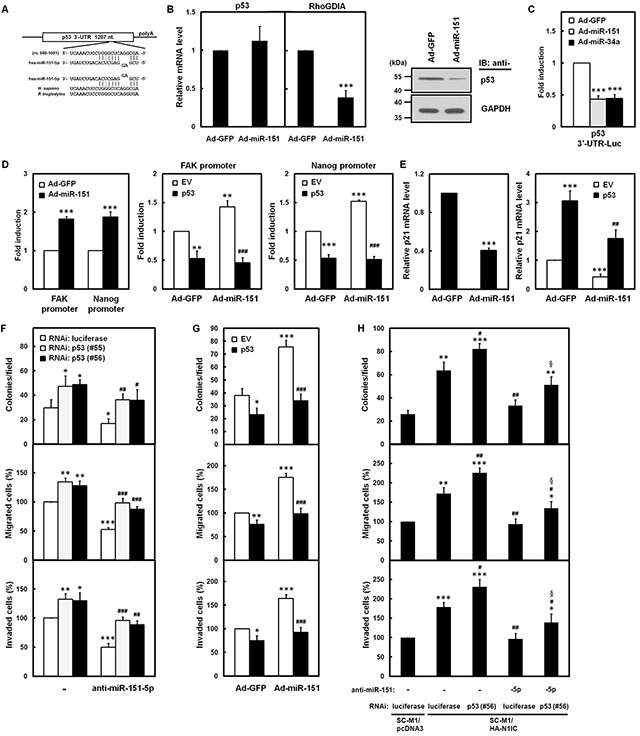
Notch1 pathway-miR-151 axis elevated aggressiveness of SC-M1 cells through down-regulation of p53 **A.** There is a putative miR-151-5p-binding site located at nucleotide 989 to 1,001 from the start of 3′-UTR of human p53 mRNA as predicted by RNAhybrid algorithm. The sequence of miR-151-5p is aligned with the 3′-UTRs of p53 in human (*H. sapiens*) and chimpanzee (*P. troglodytes*). **B.** After infection with adenoviruses expressing miR-151 (Ad-miR-151) or GFP (Ad-GFP), the transcript levels of p53 and RhoGDIA mRNAs in SC-M1 cells were measured by quantitative real-time PCR (*left*). Whole-cell extracts of the infected SC-M1 cells were prepared for Western blot analysis using anti-p53 and anti-GAPDH antibodies (*right*). **C.** After transfection with p53 3′-UTR-Luc reporter plasmid for 24 hours, SC-M1 cells were infected with adenoviruses expressing miR-151, miR-34a (Ad-miR-34a), or GFP for reporter gene assay. **D.** After transfection with reporter plasmids containing FAK or Nanog promoters for 24 hours, SC-M1 cells were infected with adenoviruses expressing miR-151 or GFP for reporter gene assay (*left*). After co-transfection with p53-expressing construct (p53) or empty vector (EV) and reporter plasmids containing FAK (*middle*) or Nanog (*right*) promoters for 24 hours, K562 cells were infected with adenoviruses expressing miR-151 or GFP for reporter gene assay. **E.** The transcript levels of p21 in SC-M1 cells were measured by quantitative real-time PCR after infection with adenoviruses expressing miR-151 or GFP (*left*). After transfection with p53-expressing construct or empty vector and subsequent infection with adenoviruses expressing miR-151 or GFP, the transcript levels of p21 in SC-M1 cells were also measured by quantitative real-time PCR (*right*). **F.** SC-M1 cells were co-transfected with antagomir-151-5p (anti-miR-151-5p) and siRNA vectors against p53 (#55 and #56) and then seeded for colony formation, migration, and invasion assays. **G.** After transfection with p53-expressing construct or empty vector, SC-M1 cells were infected with adenoviruses expressing miR-151 or GFP and then seeded for colony formation, migration, and invasion assays. **H.** The N1IC-expressing SC-M1/HA-N1IC and SC-M1/pcDNA3 control cells were co-transfected with 100 nM antagomir-151-5p or scrambled control and siRNA vectors against p53 (#56) or luciferase for colony formation, migration, and invasion assays. *, *P* <0.05; **, *P* <0.01; ***, *P* <0.001. #, *P* < 0.05; ##, *P* < 0.01; ###, *P*< 0.001. §, *P* < 0.05.

To identify potential miR-151-5p targets involve in gastric carcinogenesis, we used RNAhybrid, RNA22 v2, and mirDIP algorithms to search for human mRNAs' 3′-UTRs containing putative miR-151-5p-binding sites. *In silico* analyses revealed that a putative binding site of miR-151-5p resides at p53 3′-UTR (Figure 7A), which suggested that miR-151-5p may be a potential regulator of p53 expression. The mRNA and protein levels of p53 in miR-151-expressing adenoviruses-infected SC-M1 cells were analyzed. Overexpression of miR-151 reduced mRNA level of RhoGDIA, a miR-151 target (17), but not p53 (Figure 7B, *left*), however, the protein level of p53 was decreased in miR-151-expressing SC-M1 cells (Figure 7B, *right*).

Reporter gene activity was attenuated in SC-M1 (Figure 7C) and K562 (Supplementary Figure S6) cells infected with adenoviruses expressing miR-151 or miR-34a, a regulator of p53, after transfection with reporter plasmid containing p53 3′-UTR. These results confirmed that miR-151 can target p53 3′-UTR. Reporter gene assays or quantitative real-time PCR analyses were performed to determine the effect of miR-151 on p53 targets such as FAK [27], Nanog [28], and p21 [29]. Overexpression of miR-151 could activate FAK and Nanog promoters (Figure 7D, *left* and Supplementary Figure S7) and this promoter activation could be suppressed by a miR-151-insensitive p53-expressing construct in SC-M1 cells (Figure 7D, *middle* and *right*). Furthermore, level of p21 mRNA was decreased by miR-151 overexpression (Figure 7E, *left*) and the miR-151-mediated down-regulation of p21 mRNA expression could be restored by a miR-151-insensitive p53-expressing construct (Figure 7E, *right*).

The function of p53 on miR-151-5p-mediated gastric cancer aggressiveness was elucidated by miR-151-5p and p53 knockdown. Transfection with siRNA vectors against p53 knocked down p53 levels in SC-M1 cells (Supplementary Figure S8A). The aggressiveness, including colony formation, migration, and invasion, of SC-M1 cells was suppressed by antagomir-151-5p (Figure 7F). This suppressive effect on aggressiveness of SC-M1 cells by miR-151-5p knockdown could be restored by co-transfecting siRNA vectors against p53. The effect of ectopic p53 was determined by transfecting a p53-expression vector into SC-M1 cells already harboring miR-151-insensitive p53-expressing construct (Supplementary Figure S8B). The miR-151-enhanced aggressiveness of SC-M1 cells was suppressed by p53 overexpression (Figure 7G). Results of Figure 4 demonstrated that N1IC-enhanced aggressiveness of SC-M1 cells could be suppressed by antagomir-151-5p. This suppressive effect of antagomir-151-5p on N1IC-enhanced aggressiveness of SC-M1 cells could be partially recovered by co-transfection with the siRNA vector against p53 (Figure 7H).

## DISCUSSION

**Figure 8:**
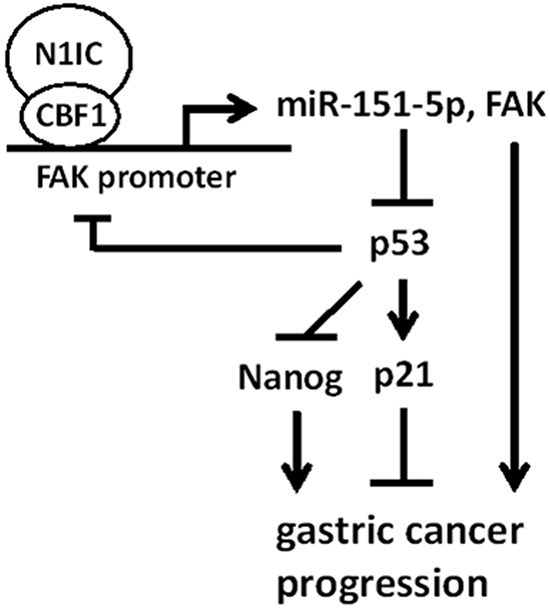
A model depicting the regulatory mechanism of Notch1/miR-151-5p/p53 axis in gastric carcinogenesis

Increasing lines of evidence reveal that Notch pathways cross-talk with miRNAs in tumorigenesis [13, 30]. This study showed that Notch1 pathway enhanced gastric carcinogenesis through miR-151-5p and FAK and Notch1/miR-151-5p/p53 axis contributed to gastric cancer progression (Figure 8). To our knowledge, this is the first report regarding the linkage of Notch1 pathway and the reciprocal regulation loop of miR-151-5p, FAK, and p53 in controlling gastric cancer progression.

Our data showed that N1IC augmented gastric cancer progression mainly through miR-151-5p (Figure 3D, 3E, and 4A). miR-151-5p also increases migration and invasion of hepatocellular carcinoma [17] and prostate cancer [31] cells. Additionally, it was found that pri- or pre-miR-151 and mature miR-151 exert different effects on separate mRNA targets [32]. Thus, miR-151 precursors could serve as not only biogenesis intermediates but also post-transcriptional regulators of miRNA activity.

The level of p53 proteins but not p53 mRNA in SC-M1 cells was suppressed by miR-151 (Figure 7B). It seems that miR-151 possibly down-regulated p53 *via* a manner of translational repression, but not mRNA degradation. The quantities of p53 protein are regulated by multiple post-translational modifications [33, 34]; however, the miR151-mediated decrement on p53 protein levels may be an indirect effect. On the other hand, p53 regulates expression and maturation of miRNAs [33], therefore, the interplay between miRNAs and p53 leads to a complex functional relationship [35]. The close interactions between miRNAs and p53 may contribute to more precise regulation of components in Notch1/miR-151-5p/p53 axis including Notch1 receptor, miR-151-5p, p53, FAK, and their down-stream targets.

It has been shown that p53 can bind to FAK promoter and then represses its activity [27], the p53 overexpression did suppress FAK promoter activity in gastric cancer cells (Figure 7D, *middle*). Activated Notch1 pathway enhanced FAK promoter activity (Figure 2) and promoted FAK and miR-151 expressions in gastric cancer cells (Figure 1). Several reports showed that Notch1 pathway interplays with p53 in regulating biological function. For example, Notch1 pathway activates p53 expression in apoptosis of neural progenitor cells [36], while p53 modulates Notch1 pathway in tumor suppression of keratinocyte [37]. Therefore, the mutual regulation loop and combinatorial control of Notch1 pathway and p53 may fine tune FAK and miR-151-5p levels in gastric cancer cells.

Our results suggested that miR-151, Notch1 receptor, and FAK may serve as potential diagnostic markers and therapeutic targets of gastric cancer. Possibly, delivery of miR-151-5p antagonists into cancer cells could restore the tumor-suppressive function of p53 and induce tumor regression for gastric cancer treatment. Further studies on Notch1/miR-151-5p/p53 axis may identify new biomarkers of diagnosis and prognosis and open a new window of chemotherapy combinations for patients with gastric malignancies.

## MATERIALS AND METHODS

### Plasmids and plasmid construction

Constructs pcDNA-HA-N1IC, pcDNA-myc-N2IC-His, pcDNA-N3IC-myc-His, and pcDNA-N4IC-myc-His contain the cDNAs encoding intracellular domains of Notch1-4 receptors, respectively [38]. Plasmids pcDNA-CBF1-myc-His [39] and pSG5Flag-RBP-VP16 [40] express CBF1 and the constitutively active RBP-Jκ mutant, respectively. Constructs RLI261AAA, EEF233AAA, and KLV249AAA direct expressions of CBF1 mutants [25]. Plasmid pCMV-p53 expresses p53 under the control of cytomegalovirus early promoter.

Luciferase reporter plasmids P-50, P-109, P-564, P-723, and P-1020 contain various lengths of FAK promoter [27]. Luciferase reporter plasmid P-1020-30 containing human FAK promoter from nucleotide −1,020 to −30 was constructed. Nanog-Luc, SOX-2-Luc, and Oct4-Luc reporter plasmids contain Nanog, SOX-2, and Oct4 promoters, respectively [41]. DNA segment of p53 3′-UTR (nucleotide 1 to 1,201) from the start of 3′-UTR was amplified by PCR from genomic DNA of SC-M1 cells to construct p53 3′-UTR-Luc reporter plasmid.

For knockdown of Notch1 receptor, FAK, and p53, their target sequences listed in the Supplementary Table S1 were constructed in the siRNA vector pLKO.1 as described [5]. miR-151 precursor sequence was amplified by PCR from genomic DNA of SC-M1 cells and then the amplified PCR product was cloned to generate a recombinant miR-151-expressing adenoviral plasmid containing a GFP tracer. All used primers for PCR are listed in the Supplementary Table S1. The constructs used in the present study were verified by sequencing.

### Cell culture and transfection

Stomach adenocarcinoma cell lines (SC-M1, AGS, AZ521, NUGC-3, KATO III, NCI-N87, and SNU-16 cells) and an erythroleukemia cell line (K562 cells) were cultured in RPMI 1640 medium containing 10% fetal bovine serum. The stable N1IC-expressing SC-M1 (SC-M1/HA-N1IC), K562 (K562/HA-N1IC), and HEK293 (HEK293/myc-N1IC) cells and control cells (SC-M1/pcDNA3, K562/pcDNA3, and HEK293/pcDNA3, respectively) were established previously [38].

Cells were transfected by electroporation or transfection reagents such as Lipofectamine™ 2000 (Invitrogen) and PolyJet™ (SignaGen). For the combination of transfection and infection, cells were transfected for 24 hours and then the transfected cells were infected with miRNA-expressing adenoviruses. After seeding onto 6-well plates, cells were transfected for subsequent luciferase reporter gene assay [42]. Luciferase activity was measured and then normalized after transfection for two days. Oligonucleotides (Ambion) of antagomir-151-5p, antagomir-151-3p, and scrambled control were transfected into cells using Lipofectamine™ 2000 [42]. The g-secretase inhibitor DAPT (Sigma-Aldrich) in dimethyl sulfoxide (DMSO) or an equal volume of DMSO were added for treatment [5].

### Recombinant adenoviruses

The recombinant miR-151-expressing adenoviral plasmid and pAdTrack-CMV control vector were used to generate recombinant adenoviruses expressing miR-151 and GFP, respectively [42]. Both infection titer and multiplicity of recombinant adenoviruses were determined following the manufacturer's protocol (Stratagene).

### Quantitative real-time PCR analysis

Total RNA was extracted by Trizol reagent (Invitrogen) and then was used to synthesize cDNA by Moloney murine leukemia virus reverse transcriptase (New England BioLabs) with an oligo (dT)_18_ primer. According to the manufacturer's protocol (Applied Biosystems), the cDNAs were amplified with primers listed in the Supplementary Table S1 using an ABI StepOne Plus system with SYBR Green Master Mix for mRNA detection [43]. For detection of mature miRNAs, cDNA synthesis was carried out by MultiScribeTM Reverse Transcriptase system and then quantitative miRNA real-time PCR was performed by the ABI StepOne Plus system (Applied Biosystems) [42].

### ChIP assay

Cells were harvested for ChIP assay using protein A-Sepharose-bound anti-Notch1 C-terminal, anti-CBF1, or anti-IgG antibodies [44]. The immunoprecipitated DNA was used for PCR amplification with the specific primers listed in the Supplementary Table S1 for the regions of FAK or HES-1 promoters.

### Western blot analysis

Whole-cell extracts were prepared and then Western blot analysis was performed with anti-Notch1 C-terminal, anti-E-cadherin, anti-plakoglobin, anti-vimentin, anti-p53 (Santa Cruz), anti-N-cadherin (BD Biosciences), anti-cleaved Notch1, anti-FAK (Cell Signaling Technology), anti-pFAK Y397 (Invitrogen), and anti-GAPDH (Biogenesis) antibodies [45].

### Cell growth and viability assays

For evaluation of cell growth and viability, the transfected and/or infected cells were seeded into 6-well plates and subsequently counted using trypan blue exclusion method. MTT assay was carried out after incubation for 24 or 48 hours and then assessed using a microplate ELISA reader (TECAN Infinite 200) as described [4].

### Colony formation, tumorsphere formation, migration, and invasion assays

The transfected and/or infected cells were seeded in soft agar for 14 days to survey the ability of anchorage-independent growth by colony formation assay [46]. After staining with crystal violet, the colonies larger than 0.1 mm in diameter were counted from 10 random fields under the microscope. For assay of tumorsphere formation, the treated cells were seeded onto 96-well ultra-low attachment plates (Corning) containing stem cell-selective medium for 9 days [42]. Number of spheres larger than 50 μm was counted under the microscope. Abilities of cellular migration and invasion were surveyed in 24-well plates for 12 or 20 hours, respectively [5]. After fixing with methanol and staining with crystal violet, numbers of the migrated or invaded cells were counted from 10 random fields under the microscope.

### *In vivo* xenografted tumorigenicity and tail vein metastasis assays

All animal experiments and protocols in this study were performed with the approval of the institutional ethical committee (Institutional Animal Care and Use Committee of National Yang-Ming University). Both xenografted tumorigenicity and tail vein metastasis assays were performed as described [5, 45]. Briefly, the treated cells were subcutaneously injected into 5-week-old BALB/c nu/nu mice purchased from National Science Council Animal Center (Taipei, Taiwan) for xenografted tumorigenicity assay. Volume of xenografts was examined and recorded every 3 days. For tail vein metastasis assay, the treated cells were injected into 6-week-old female NOD-SCID mice (National Taiwan University, Taipei, Taiwan) by tail vein injection [45]. Count of metastatic nodules in lung was measured by gross and microscopic examination after sacrificing.

### Surgical samples

Tissues of gastric adenocarcinoma were obtained from gastric cancer patients who had undergone gastric surgical resection at the Department of Surgery, Taipei Veterans General Hospital. Prior to surgery, none of these patients had undergone chemotherapy or radiotherapy. The informed consent was obtained from all patients prior to study and the analysis of tissue specimen was also approved by the Institutional Review Board in Taipei Veterans General Hospital.

### Statistical analyses

Statistical analysis was carried out by an independent Student's t-test for simple comparison of two groups. Level of statistical significance was set at *P* value less than 0.05 for all tests.

## SUPPLEMENTARY FIGURES AND TABLE



## Abbreviations

N1IC: Notch1 receptor intracellular domain
miR-151: microRNA-151
FAK: focal adhesion kinase
EMT: epithelial-mesenchymal transition
MTT: 3-(4,5-dimethyl-2-thiazolyl)-2,5-diphenyl tetrazolium bromide
3′-UTR: 3′-untranslated region
TCGA: The Cancer Genome Atlas
HCC: hepatocellular carcinoma
