# Supplementary material for: Notch1 pathway-mediated microRNA-151-5p promotes gastric cancer progression
Source: Oncotarget. 2016 May 13;7(25):38036–51. doi: 10.18632/oncotarget.9342 (PMC5122370; doi:10.18632/oncotarget.9342)
Supplement: Supplementary file 1 [file oncotarget-07-38036-s001.pdf]

SUPPLEMENTARY FIGURES AND TABLE

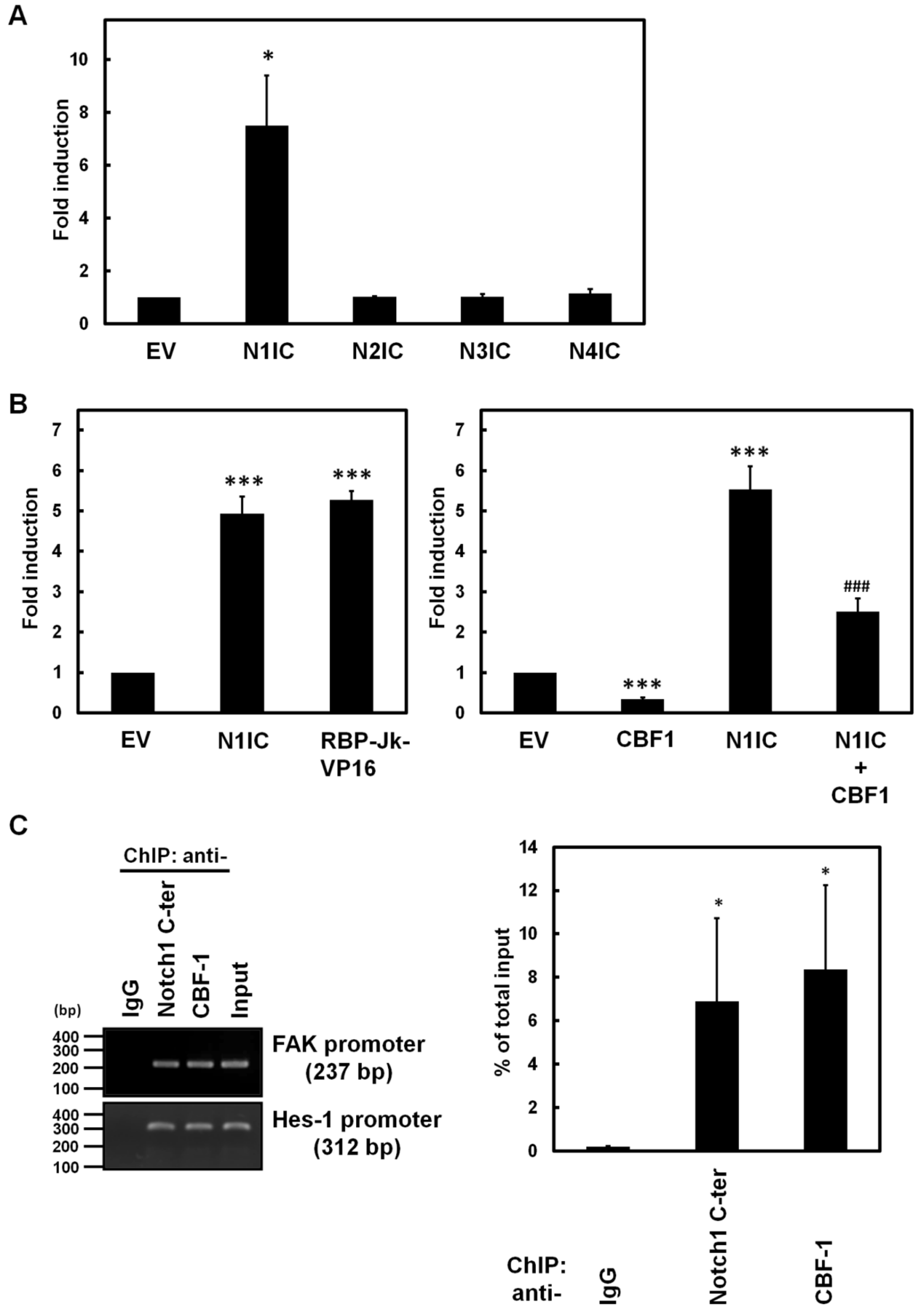

Supplementary Figure S1: Activated Notch1 pathway induced FAK promoter activity in K562 cells. A. As described in the legend to Figure 2B, reporter plasmid P-1020 was co-transfected with expression plasmids of Notch1 receptor (N1IC), Notch2 receptor (N2IC), Notch3 receptor (N3IC), and Notch4 receptor (N4IC) intracellular domains or empty vector (EV) into K562 cells for reporter gene assay. **B.** As described in the legend to Figure 2D, reporter plasmid P-1020 was co-transfected with expression constructs of N1IC and constitutively active RBP-Jk-VP16 fusion protein (*left*) or CBF1 (*right*) into K562 cells for reporter gene assay. **C.** As described in the legend to Figure 2F, K562/HA-N1IC cells were harvested for ChIP assay using anti-IgG, anti-Notch1 C-ter, and anti-CBF1 antibodies. *, *P* <0.05; ***, *P* <0.001. ###, *P*< 0.001.

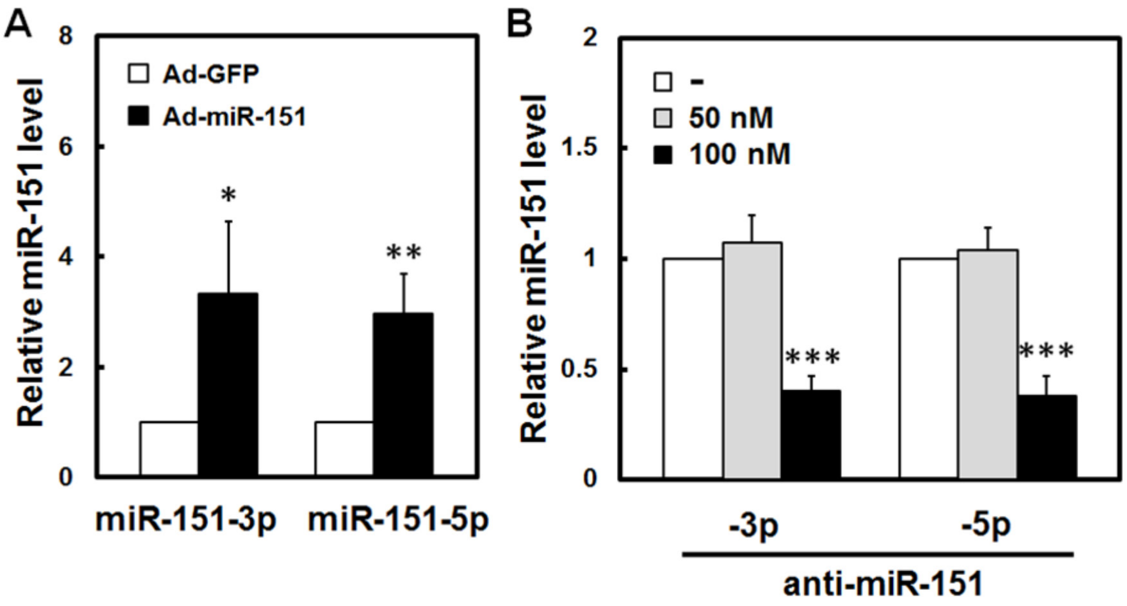

Supplementary Figure S2: The miR-151 in SC-M1 gastric cancer cells was overexpressed and knocked down after infection with miR-151-expressing adenoviruses and transfection with antagomir-151, respectively. A. The relative levels of miR-151-3p and miR-151-5p in SC-M1 cells infected with adenoviruses expressing miR-151 (Ad-miR-151) or GFP (Ad-GFP) were measured by miRNA quantitative real-time PCR. The levels of miR-151-3p and miR-151-5p in the cells infected with adenoviruses expressing GFP were set to unity. **B.** SC-M1 cells were transfected with 50 or 100 nM antagomir-151-3p (anti-miR-151-3p), antagomir-151-5p (anti-miR-151-5p), and scrambled control (-) for 48 hours. The relative levels of miR-151-3p and miR-151-5p in the transfected cells were measured by miRNA quantitative real-time PCR. *, $P < 0.05$; **, $P < 0.01$; ***, $P < 0.001$.

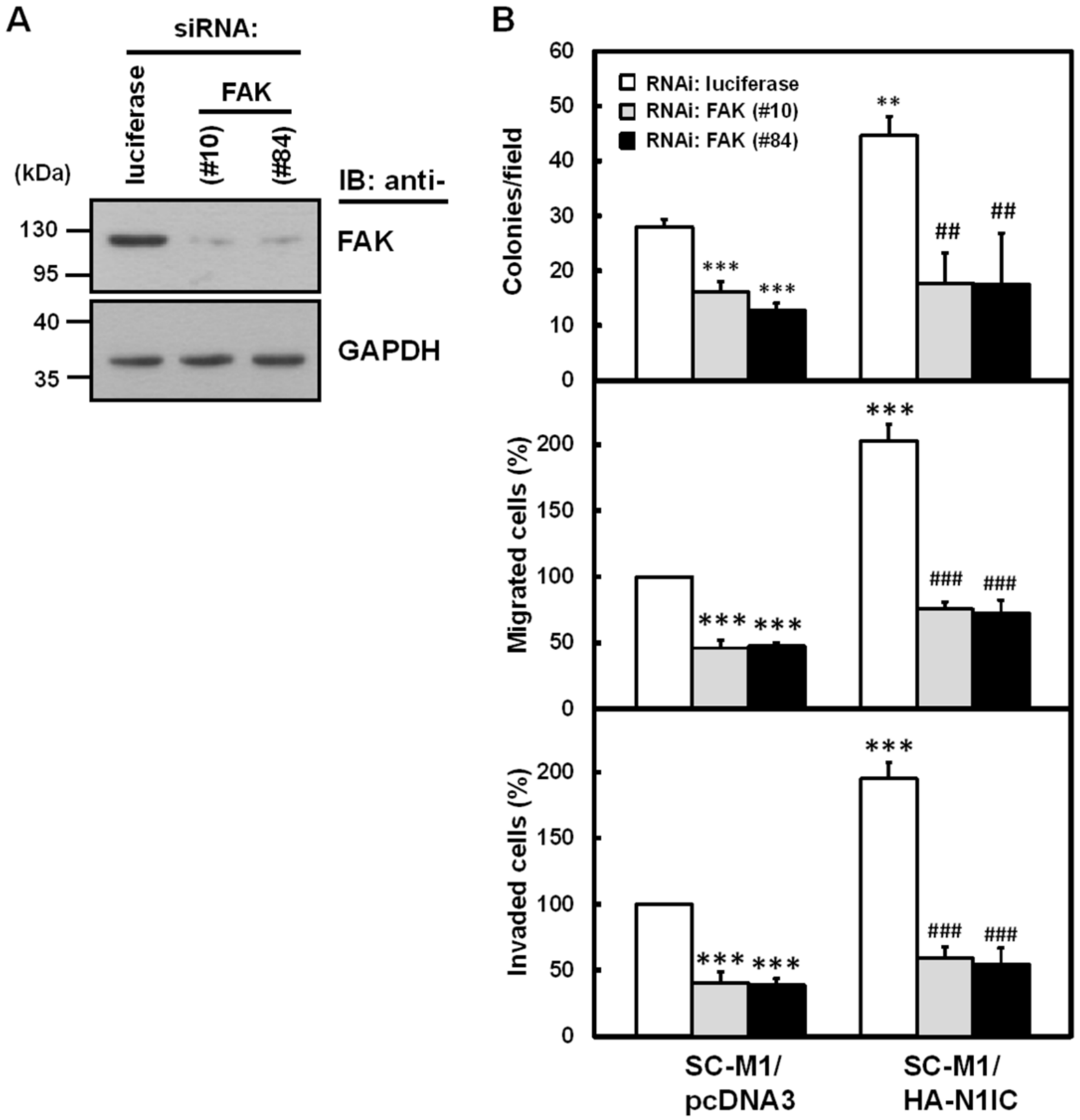

Supplementary Figure S3: Activated Notch1 pathway promoted gastric cancer progression through FAK. A. After transfection with siRNA vectors against FAK (#10 and #84) or luciferase into SC-M1 cells for 48 hours, whole-cell extracts of the transfected cells were prepared for Western blot analysis using anti-FAK and anti-GAPDH antibodies. **B.** After transfection with siRNA vectors against FAK (#10 and #84) or luciferase into N1IC-expressing SC-M1/HA-N1IC cells and SC-M1/pcDNA3 control cells for 48 hours, the transfected cells were seeded for colony formation (*upper*), migration (*middle*), and invasion (*lower*) assays. **, P <0.01; ***, P <0.001. ##, P < 0.01; ###, P< 0.001.

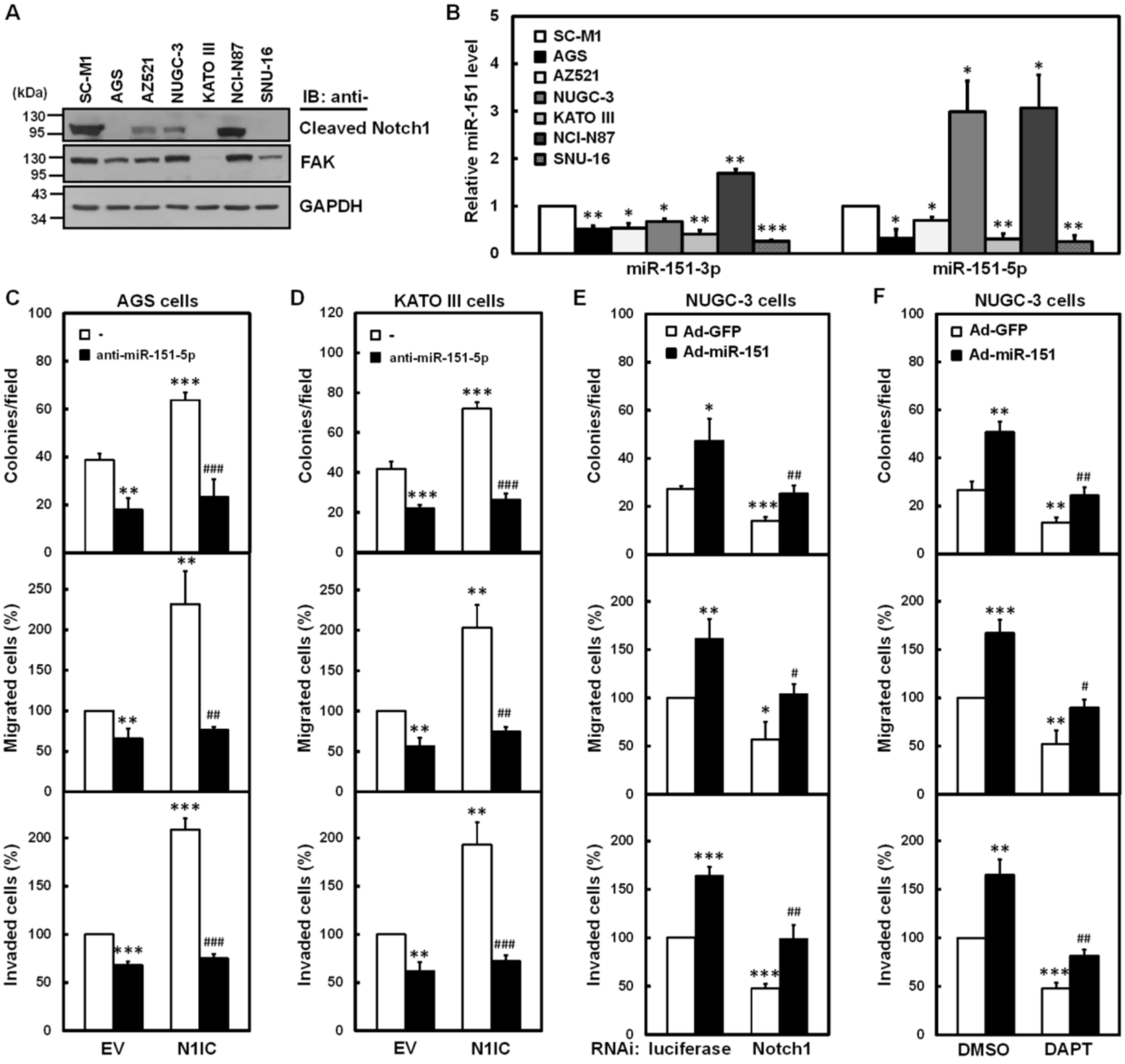

Supplementary Figure S4: N1IC enhanced gastric cancer progression of AGS, KATO III, and NUGC-3 cells through miR-151-5p. **A.** Whole-cell extracts of SC-M1, AGS, AZ521, NUGC-3, KATO III, NCI-N87, and SNU-16 cells were prepared for Western blot analysis using anti-cleaved Notch1, anti-FAK, and anti-GAPDH antibodies. **B.** The relative levels of miR-151-3p and miR-151-5p in SC-M1, AGS, AZ521, NUGC-3, KATO III, NCI-N87, and SNU-16 cells were measured by miRNA quantitative real-time PCR. **C, D.** AGS (C) or KATO III (D) cells were co-transfected with expression construct of Notch1 receptor intracellular domain (N1IC) or empty vector (EV) and 100 nM antagomir-151-5p (anti-miR-151-5p) or scrambled control (–) and seeded for subsequent colony formation (*upper*), migration (*middle*), and invasion (*lower*) assays. **E.** After transfection with siRNA vectors against Notch1 receptor or luciferase, NUGC-3 cells were infected with adenoviruses expressing miR-151 (Ad-miR-151) or GFP (Ad-GFP) and then seeded for colony formation (*upper*), migration (*middle*), and invasion (*lower*) assays. **F.** After infection with adenoviruses expressing miR-151 or GFP, NUGC-3 cells were treated with 50 mM DAPT or an equal volume of DMSO and seeded for subsequent colony formation (*upper*), migration (*middle*), and invasion (*lower*) assays. *, P <0.05; **, P <0.01; ***, P <0.001. #, P < 0.05; ##, P < 0.01; ###, P< 0.001.

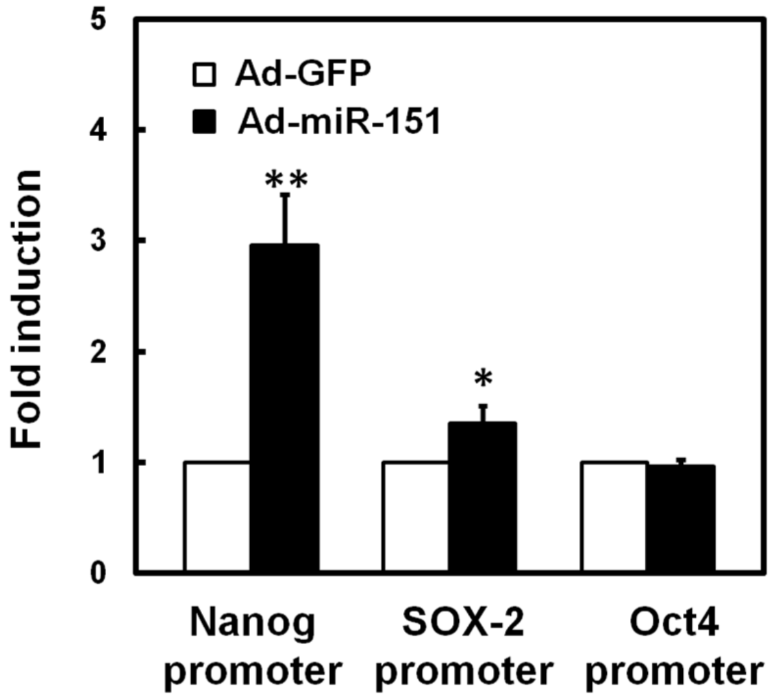

Supplementary Figure S5: miR-151 elevated activities of Nanog and SOX-2 promoters in K562 cells. As described in the legend to Figure 5C, K562 cells transfected with reporter plasmids containing Nanog, SOX-2, and Oct4 promoters were infected with adenoviruses expressing miR-151 or GFP for reporter gene assay. *, $P < 0.05$; **, $P < 0.01$.

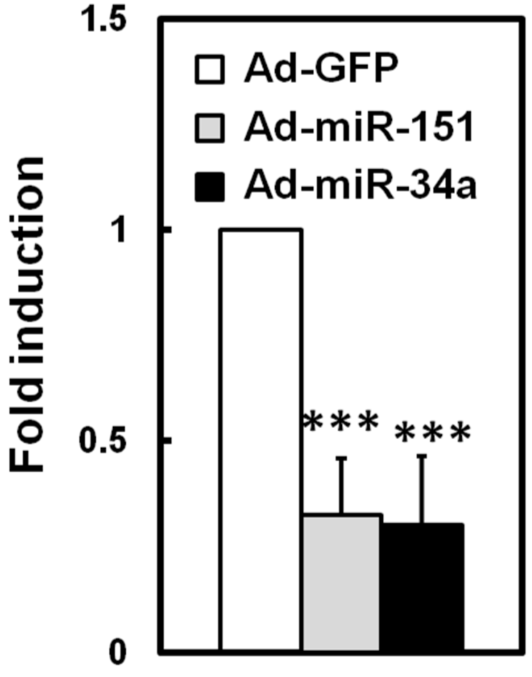

Supplementary Figure S6: miR-151 suppressed activity of reporter gene containing p53 3′-UTR in K562 cells. As described in the legend to Figure 7C, K562 cells transfected with p53 3′-UTR-Luc reporter plasmid were infected with adenoviruses expressing miR-151, miR-34a (Ad-miR-34a), or GFP for reporter gene assay. ***, $P < 0.001$.

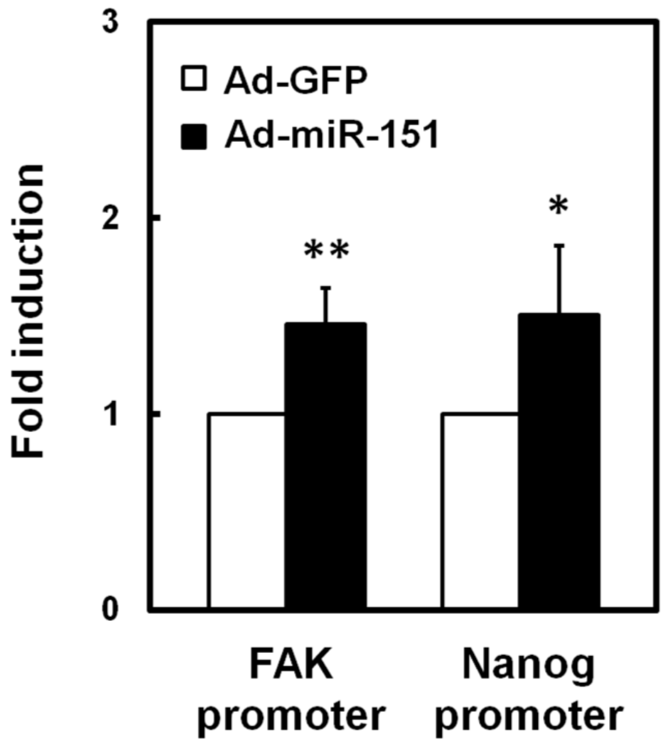

Supplementary Figure S7: miR-151 enhanced activities of FAK and Nanog promoters in K562 cells. As described in the legend to Figure 7D, K562 cells transfected with reporter plasmids containing FAK or Nanog promoters were infected with adenoviruses expressing miR-151 or GFP for reporter gene assay. *, *P* <0.05; **, *P* <0.01.

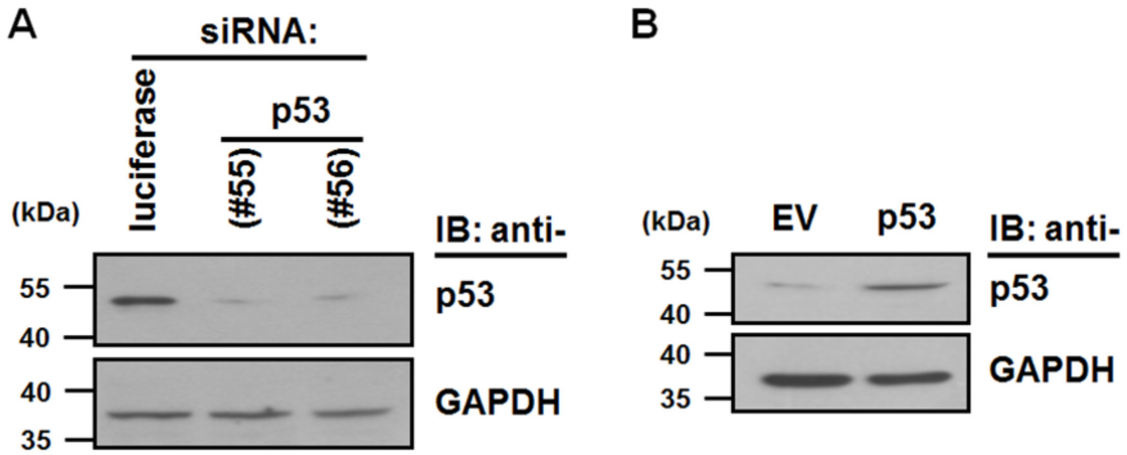

Supplementary Figure S8: The p53 in SC-M1 gastric cancer cells was knocked down and overexpressed after transfection with siRNA vectors against p53 and p53-expressing construct, respectively. A. After transfection with siRNA vectors against p53 (#55 and #56) or luciferase into SC-M1 cells, whole-cell extracts of the transfected cells were prepared for Western blot analysis using anti-p53 and anti-GAPDH antibodies. **B.** After transfection with p53-expressing construct (p53) or empty vector (EV) into SC-M1 cells, whole-cell extracts of the transfected cells were prepared for Western blot analysis using anti-p53 and anti-GAPDH antibodies.

Supplementary Table S1: Sequence of primers for siRNA, PCR, and real-time PCR

Assays		Sequence (5′ to 3′)	Amplicon (bp)
siRNA	Notch1 receptor	GCCGAACCAATACAACCCTCT	
	FAK (#10)	GATGTTGGTTTAAAGCGATTT	
	FAK (#84)	CAACAGGTGAAGAGCGATTAT	
	p53 (#55)	GTCCAGATGAAGCTCCCAGAA	
	p53 (#56)	CACCATCCACTACAACTACAT	
PCR	miR-151	F ATAAGATCTGGCTGCAACCCGTGTTC<br>R ATAGTCGACCTCAGTGGAGCATTCC	324
	p53 3′-UTR	F GAATTCGCCTGACTCAGACTGACATTC<br>R GAATTCTCAGACACACAGGTGGCA	1,217
real-time PCR	CD44	F TCCAACACCTCCCAGTATGACA<br>R GGCAGGTCTGTGACTGATGTACA	83
	Nanog	F CCTGTGATTTGTGGGCCTG<br>R GACAGTCTCCGTGTGAGGCAT	78
	Oct4	F GGTGGAGGAAGCTGACAACAA<br>R AAATTCTCCAGGTTGCCTCTCA	123
	SOX-2	F GTATCAGGAGTTGTCAAGGCAGAG<br>R TCCTAGTCTTAAAGAGGCAGCAAAC	78
	p53	F CCCAAGCAATGGATGATTTGA<br>R GGCATTCTGGGAGCTTCATCT	91
	RhoGDIA	F GTTTGTGCTGAAGGAGGGTGT<br>R TCGTCTGTGAAGCGGGACT	249
	p21	F CAGGGGACAGCAGAGGAAGA<br>R TTAGGGCTTCCTCTTGGAGAA	189
	GAPDH	F AAATCCCATCACCATCTTCC<br>R TCACACCCATGACGAACA	194
ChIP	FAK promoter (P-50)	RVprimer3 CTAGCAAAATAGGCTGTCCC<br>GLprimer2 CTTTATGTTTTTGGCGTCTTCCA	299
	FAK promoter (chromosomal DNAs and P-1020-30)	F TTCTCCAGAGGGGACCCCTTC<br>R CGTAATTTGTCCTGTAGTGACC	237
	Hes-1 promoter (chromosomal DNAs)	F CAAGACCAAAGCGGAAAGAA<br>R GGATCCTGTGTGATCCCTAGGC	312
